# Studies on Liquefaction Time and Proteins Involved in the Improvement of Seminal Characteristics in Dromedary Camels (*Camelus dromedarius*)

**DOI:** 10.1155/2016/4659358

**Published:** 2016-02-18

**Authors:** Gorakh Mal, Sumant Vyas, Alagiri Srinivasan, Nitin Vasant Rao Patil, Krishan Murari Lal Pathak

**Affiliations:** ^1^National Research Centre on Camel, Bikaner, Rajasthan 334 001, India; ^2^Indian Veterinary Research Institute, Regional Station, Palampur 176 061, India; ^3^Department of Biophysics, All India Institute of Medical Sciences, New Delhi 110 029, India; ^4^Indian Council of Agricultural Research (ICAR), Krishi Bhavan, New Delhi 110 001, India

## Abstract

Semen was collected from six dromedary camels using artificial vagina during rutting season. Liquefaction of the viscous semen occurred in 23.89 ± 1.49 h. During liquefaction, proteins with molecular masses of 24.55 kDa and 22.07 kDa appeared in conjunction with the disappearance of intact 26.00 kDa protein after 18–24 h. These proteins were identified as *β*-nerve growth factors (*β*-NGFs) in liquefied camel semen. Guanidine-HCL improves the rheological characteristics of dromedary camel semen along with significant (*P* < 0.01) increase in sperm motility. No significant differences were found in viability of spermatozoa indicating no visible detrimental effects on spermatozoa. The cause of semen viscosity, as well as proteins that are present in liquefied dromedary camel seminal plasma, is described for the first time.

## 1. Introduction

Earlier studies carried out on semen evaluation revealed that dromedary camel's spermatozoa are densely clustered and the heads of spermatozoa are embedded and tightly secured and some process of liquefaction of semen coagulum releases spermatozoa in batches, which develop progressive motility [1]. Dromedary camel semen is highly viscous in nature, exhibits low progressive motility, and liquefies slowly. The viscous seminal plasma is currently the major hurdle for the development of assisted reproductive technologies (ARTs) in dromedary camels. Due to viscosity of the ejaculated semen, AI technology in dromedary camels is lacking, which needs to be liquefied before its evaluation [2]. It is necessary to discover the cause of the viscosity and gain an understanding of the role of seminal plasma components in sperm function and fertility. Absence of sperm motility has also been reported [3–5]. These workers have emphasized investigating the factors responsible for the lack of sperm motility in camel semen. No systematic study has been carried out so far regarding the coagulum formation in camel semen and factors responsible for its liquefaction. Higher concentrations of calcium (Ca), zinc (Zn), and iron (Fe) might play an important role in the process of coagulation and liquefaction of dromedary camel semen [6]. Differential protein profiles expression was observed in dromedary seminal plasma [7]. The process of coagulation and liquefaction has been studied in the rat [8, 9], guinea pig [10], and human [11–16]. Following ejaculation in the human, a soft clot is formed that then dissolves over 5–20 min [17]. Following dissolution of the clot, a postliquefaction period ensues during which further proteolytic degradation occurs to produce low molecular mass proteins [18]. The major structural components of human semen coagulum have been described as disulfide-linked complexes known as SgI and SgII. Further, it was found that SgI complexes have the property to block the sperm motility. In human, SgI and SgII are secreted form seminal vesicles, which are found to be absent in camel. In the case of dromedary semen, the spermatozoa are entrapped in a fibrinous network and thus it needs to be liquefied before the spermatozoa are free and the sample becomes homogenous. Collagenase, fibrinolysin, hyaluronidase, and trypsin have all been used to reduce the viscosity of camelid semen; however, all the enzymes have been seen to cause acrosomal damage in spermatozoa [3]. Semen liquefies with collagenase [2] but there was reduced motility of spermatozoa after its dilution with different extenders and storage at room or refrigeration temperature indicating that the enzymes do have some detrimental effects on the spermatozoa. Collagenase type I improved the rheological characteristics of dromedary camel semen [19].

The objectives of this study were to know the liquefaction time, identification of the proteins involved in this process, and improvement of rheological characteristics of camel semen.

## 2. Materials and Methods

### 2.1. Semen Collection and Preparation of Seminal Plasma

The study was conducted on 6 Jaisalmeri breed camels (*Camelus dromedarius*) belonging to the National Research Centre on Camel, Bikaner, India. The animals were apparently healthy and were maintained under uniform standard conditions of feeding and management. The research program was approved by the institute research and ethical committee. Semen samples were collected twice a week using artificial vagina during rutting season (December to March) as described previously [4].

### 2.2. Time Course Study of the Liquefaction

Two time course studies were carried out to know the liquefaction time at refrigerated temperature. Samples were divided into 2 parts. One portion of ejaculate was evaluated for the changes in semen rheological characteristics by pipetting semen at different time intervals. No thread formation in semen was considered to be liquefied semen. The other portion was centrifuged at 6000 rpm for 30 min [20] and aliquots of seminal plasma were taken out at different time intervals to compare the protein profiles of fresh (i.e., clotted) versus aliquoted samples (i.e., liquefied samples). Cocktail of protease inhibitor (100 *μ*L protease inhibitor cocktail used/mL of seminal plasma, Sigma-Aldrich, USA, catalogue number P8340) was added in aliquoted samples to inhibit the proteolytic activity and stored at −196°C until it was used. Protein concentrations for each time point were determined and samples were processed for SDS-PAGE.

### 2.3. One-Dimensional Sodium Dodecyl Sulfate-Polyacrylamide Gel Electrophoresis (1D SDS-PAGE)

Protein concentration was determined using the Pierce BCA protein quantification assay (Pierce, Rockford, IL, USA) according to the manufacturer's instructions. Seminal plasma samples were reduced in Laemmli buffer and SDS-polyacrylamide gel electrophoresis of seminal plasma proteins was carried out [21]. The proteins on gels were visualized by Coomassie Brilliant Blue G-250 (Sigma-Aldrich, St. Louis, Missouri, USA). The molecular weight of the proteins was estimated by using reference protein molecular weight markers: rabbit muscle myosin (205.0 kDa), phosphorylase b (97.4 kDa), bovine serum albumin (66.0 kDa), oval albumin (43.0 kDa), carbonic anhydrase (29.0 kDa), and lysozyme (14.3 kDa).

### 2.4. Mass Spectrometry, MALDI-TOF/MS Analysis and Identification of *β*-NGF

The protein bands (24.55 and 22.07 kDa) were excised from Coomassie stained SDS-PAGE gel. Mass spectrometry and MALDI-TOF/MS were carried out [22]. The analysis was performed using a Bruker autoflex MALDI-TOF/MS (Bruker Daltonics) system equipped with 355 nm ND: YAG laser. The combined MS and MS/MS spectra were deposited to MASCOT version 2.1 (Matrix Science, London, UK) using GPS Explorer software version 3.6 (Applied Biosystems). The number of identified peptides, sequence coverage, and number of MS/MS identified peptides were taken during protein identification.

### 2.5. Effect of Denaturing Agents on Semen Rheological Characteristics

36 ejaculates from 6 camels were used to study the effect of various denaturing agents for improving the seminal characteristics. Each ejaculate was divided into 3 parts. One part served as control and the remaining two parts were incubated either with buffer containing 20 mM TRIS + 8 M Urea + 33 mM DTT or with 2.5 M guanidine-HCL at 37°C for 5–30 min for disruption of seminal clot and for the induction of liquefaction. Liquefaction of the semen was assessed by pipetting the semen as described above. Percentages of sperm motility and viability were determined. At least 200 spermatozoa were counted for each group. Sperm viability (%) was assessed with Eosin-Nigrosin stain. Sperm motility and viability were analyzed using *t*-test [23].

## 3. Results

### 3.1. Liquefaction of Camel Semen

Complete liquefaction was observed to be 23.89 ± 1.49 h varying from 18 to 41 h.

### 3.2. Protein Profiles in Liquefied Semen and Identification of *β*-Nerve Growth Factors in Liquefied Semen

**Figure 1:**
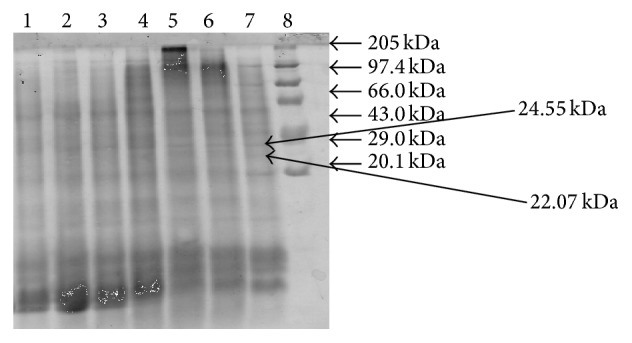
Protein profiles of fresh and liquefied semen. Lane 1, 0 hours; lane 2, 6 h; lane 3, 12 h; lane 4, 18 h; lane 5, 24 h; lane 6, 30 h; lane 7, 36 h; and lane 8, protein markers.

**Figure 2 fig2:**
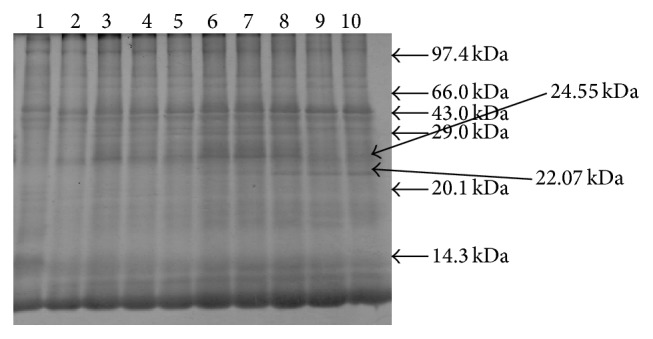
Protein profiles of fresh and liquefied semen. Lane 1, 0 hours; lane 2, 6 h; lane 3, 12 h; lane 4, 18 h; lane 5, 24 h; lane 6, 30 h; lane 7, 36 h; lane 8, 42 h; lane 9, 48 h; and lane 10, 72 h.

**Figure 3 fig3:**
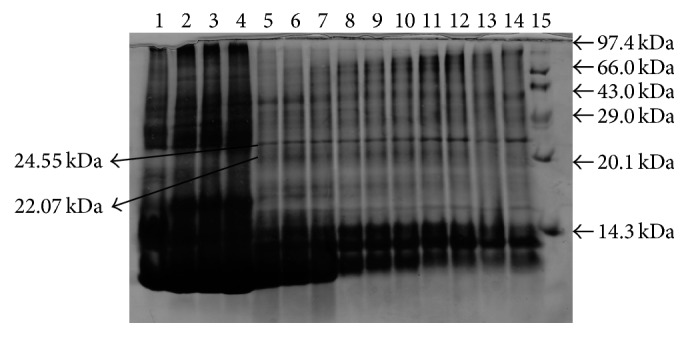
Protein profiles of fresh and liquefied semen. Lane 1, 0 hours; lane 2, 6 h; lane 3, 12 h; lane 4, 18 h; lane 5, 24 h; lane 6, 30 h; lane 7, 36 h; lane 8, 42 h; lane 9, 48 h; lane 10, 54 h; lane 11, 60 h; lane 12, 66 h; lane 13, 72 h; lane 14, 84 h; and lane 15, protein markers.

**Figure 4:**
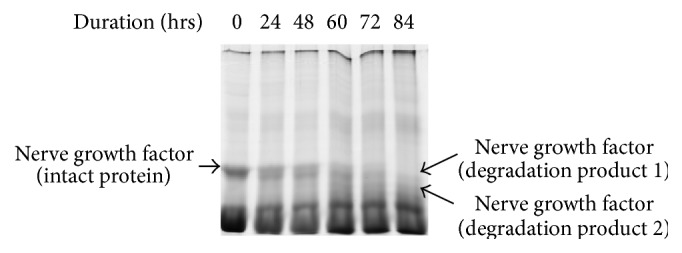
SDS-PAGE for identification of *β*-nerve growth factor in camel semen. Lane 1, 0 hours; lane 2, 24 h; lane 3, 48 h; lane 4, 60 h; lane 5, 72 h; and lane 6, 84 h.

12 protein bands were observed in unliquefied semen and 13 bands were observed in liquefied semen. ~26.00 kDa protein bands were found in fresh seminal plasma and remained stable up to 18–24 h (as shown in Figures 1 [Fig fig2] [Fig fig3]–4) with individual animal to animal variations (even up to 41 h) and after that these proteins start to degrade as the camel semen starts to liquefy. 26.00 kDa proteins bands have been identified as nerve growth factor. Proteins with molecular masses of 24.55 kDa and 22.07 kDa appeared in conjunction with the disappearance of intact 26.00 kDa protein after 18–24 h. These bands were characterized by mass spectrophotometry; upper and lower bands were identified as degraded nerve growth factors (Table 1).

### 3.3. Effect of Denaturing Agents in Liquefaction

It was observed that initial motility was found to be nearly zero and spermatozoa were entrapped in the gel so the estimation of motility was not possible. No visible effect on seminal clot was noticed with buffer. However, guanidine-HCL showed a partial disruption of seminal clot after 30 min as frequency of thread formation reduced significantly. Thread formation was observed in all freshly ejaculated semen, 77.78% thread formation was observed in buffer treated semen, and 41.67% was observed in guanidine-HCL treated semen after 30 minutes (Table 2). Initial individual motility in control semen was found to be nil. A significant higher (*P* < 0.01) motility was observed for the semen samples treated with guanidine-HCL compared to buffer treated and control group. No significant differences were found in viability of spermatozoa (Table 2) indicating no visible detrimental effects on spermatozoa.

## 4. Discussion

Hyperviscous nature of camel semen may have advantages in preventing sperm losses. The time of liquefaction of semen in dromedary camels was 4.5 to 9.6 min [24] and 8 to 48 h in alpaca camels [25], while a liquefaction time of 23.89 ± 1.49 h was found in dromedary camel semen in the present study.

In human semen hyperviscous seminal plasma may cause asthenozoospermia [26]. The existence of a highly organized network of disulfide bonds, oligosaccharide and peptide chains in seminal plasma, responsible for the rheological characteristics of ejaculates with high viscosity was suggested [27]. These molecules, which would be responsible for the hyperviscous rheological behavior, could be a key factor in sperm physiology and, therefore, in sperm motility.

The electrophoretic patterns of camel seminal plasma revealed the presence of 10 to 14 protein fractions [24].

*β*-nerve growth factor in fresh ejaculated semen of dromedary camel was identified [22]. During time course study, it was observed that this protein undergoes fragmentation to yield 2 bands of 24.55 kDa and 22.07 kDa approximately in size in liquefied semen. These proteins were identified as *β*-nerve growth factors. *β*-NGFs appeared to remain in the liquefied semen up to 84 h (i.e., the duration up to which experiment was conducted).

Enzymatic treatments used in bactrian and alpaca semen have decreased the viscosity but deleterious effects on spermatozoa were observed [28–33]. In our study, reduction in thread formation along with significant increase in motility was observed in guanidine-HCL treated samples. An increase in sperm motility might be due to guanidine-HCL by modifying semen rheological characteristics. Effect of different extenders (Citrate yolk, Tris fructose yolk, Androhep, Laciphos, Green Buffer) on the semen viscosity and sperm viability of the dromedary camel was studied [34] and a beneficial effect of adding extenders on liquefaction of camel semen and sperm motility was found. No posttreatment deleterious effect was observed on sperm viability. 0.1% collagenase was reported to be improving the rheological characteristics of dromedary camel semen [19].

## 5. Conclusions

Time course studies on liquefaction indicated protein transformation and a definite role of *β*-NGF in the process of liquefaction. *β*-NGF proteins appeared from 24 h onwards and remain up to 84 h in liquefied semen. Recently, experiments carried out by different workers revealed that nerve growth factors are also involved in ovulation induction in alpaca camels. The results obtained in this study show that treatment with 2.5 M guanidine-HCL provides a way to enhance dromedary semen rheological characteristics, permitting the separation of spermatozoa from seminal plasma and promoting sperm progressive motility. It will be of pertinent interest to study the sperm interactions of the liberated cleavage products of the unfragmented protein. The role of seminal plasma proteins in sperm function in dromedary camels requires further research.

## Figures and Tables

**Table 1:** Proteins identified in dromedary seminal plasma by MALDI-TOF/MS of the 24.55 and 22.07 kDa bands.

Bands position	Protein name	Accession number	Number of peptides	Sequence coverage	Peptide sequences	Database search
Upper	*β*-nerve growth factor precursor	gi/2499187	2	12.7	EVMVLGEVNINNSVFKQYFFETK	NCBInr

Lower	*β*-nerve growth factor	gi/73981127	2	4.6	GKEVMVLGEVNINNSVFKQYFFETK	NCBInr

**Table 2:** Rheological characteristics, motility (%), and live sperm (%) in control, buffer, and guanidine-HCL treated camel semen.

Groups	Thread formation (%) at different time intervals			Motility (%) at different time intervals			Live sperm (%) at different time intervals		
	0 min	15 min	30 min	0 min	15 min	30 min	0 min	15 min	30 min
Control	100	100	100	0	0	0	80.66 ± 1.10	80.61 ± 1.15	79.80 ± 1.21
Buffer	100^a^	86.11^b^	77.78^c^	0^a^	12^b^ ± 1.32	15^b^ ± 1.26	80.66 ± 1.10	79.88 ± 1.12	79.50 ± 1.09
Guanidine-HCL	100^a^	72.22^b^	41.67^c^	0^a^	25^b^ ± 1.41	40^c^ ± 1.52	80.66 ± 1.10	80.33 ± 1.27	78.68 ± 1.22

^a,b,c^Superscripts denote significant differences (*P* < 0.01) in a row for corresponding parameter.
